# Advanced glycation end-products, cardiac function and heart failure in the general population: The Rotterdam Study

**DOI:** 10.1007/s00125-022-05821-3

**Published:** 2022-11-08

**Authors:** Banafsheh Arshi, Jinluan Chen, M. Arfan Ikram, M. Carola Zillikens, Maryam Kavousi

**Affiliations:** 1 Department of Epidemiology, Erasmus MC, University Medical Center Rotterdam, Rotterdam, the Netherlands; 2 Department of Internal Medicine, Erasmus MC, University Medical Center Rotterdam, Rotterdam, the Netherlands

**Keywords:** Advanced glycation end-products, Cardiac function, Diabetes, Heart failure, Left ventricular diastolic function, Left ventricular systolic function, Sex differences, Skin autofluorescence

## Abstract

**Aims/hypothesis:**

The aim of this work was to assess the association of advanced glycation end-products (AGEs), measured by skin autofluorescence (SAF), with prevalent heart failure, and with systolic and diastolic cardiac function, in a large population-based cohort study.

**Methods:**

We assessed the cross-sectional association between SAF and prevalent heart failure among 2426 participants from the population-based Rotterdam Study, using logistic regression. Next, among individuals free of heart failure (*N*=2362), we examined the link between SAF (on a continuous scale) and echocardiographic parameters of left ventricular (LV) systolic and diastolic function using linear regressions. Analyses were adjusted for traditional cardiovascular risk factors.

**Results:**

Higher levels of SAF were associated with higher odds of prevalent heart failure (multivariable adjusted OR 2.90 [95% CI 1.80, 4.62] for one unit higher SAF value). Among individuals without heart failure, one unit increase in SAF was associated with 0.98% lower LV ejection fraction (mean difference [β] −0.98% [95% CI −1.45%, −0.50%]). The association was stronger among participants with diabetes (β −1.84% [95% CI −3.10%, −0.58%] and β −0.78% [95% CI −1.29%, −0.27%] among participants with and without diabetes, respectively). Associations of SAF with diastolic function parameters were not apparent, except in men with diabetes.

**Conclusions/interpretation:**

AGE accumulation was independently associated with prevalent heart failure. Among individuals free of heart failure, AGEs were associated with cardiac function, in particular systolic function. This association was present in participants with and without diabetes and was more prominent in those with diabetes.

**Graphical abstract:**

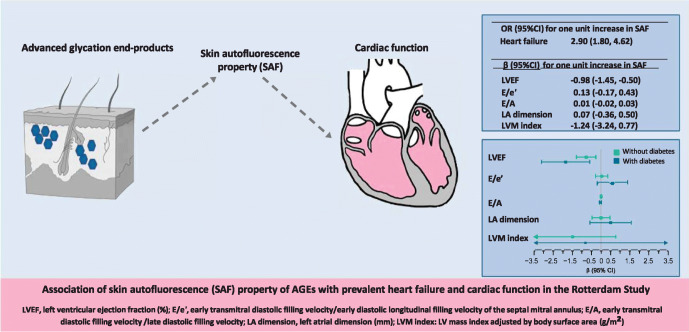

**Supplementary Information:**

The online version of this article 10.1007/s00125-022-05821-3 contains peer-reviewed but unedited supplementary material.

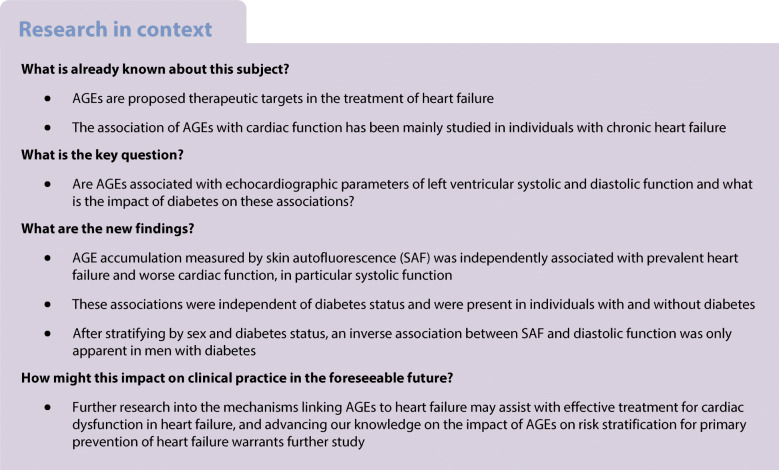



## Introduction

Heart failure is a global epidemic with increasing occurrence particularly in the elderly [1]. It is a syndrome arising from structural or functional cardiac abnormalities, leading to systolic and/or diastolic ventricular dysfunction [1, 2]. Advanced glycation end-products (AGEs) are formed after non-enzymatic reactions between protein and sugar residues that accumulate in the human body [3]. Their formation occurs through processes such as cooking and cigarette smoking [4]. AGEs accumulate throughout the body in plasma and in vessels, skin and cardiac tissue [3, 4]. The extent of non-enzymatic glycation depends on glucose concentration and time of exposure [5]. AGEs accumulate continuously on wall proteins of vessels and also in other tissues with ageing and are enhanced among individuals with diabetes, leading to complications such as diabetes microangiopathy [5]. These end-products have also been associated with CVD and increased mortality risk in the general population [6–8].

An association between AGEs and heart failure after acute decompensation has recently been reported [9]. AGEs may contribute to heart failure via induction of cardiac and vascular dysfunction. Mainly, glycation of the extracellular matrix proteins within the vasculature and subsequent irreversible cross-linking of those proteins may lead to increasing arterial stiffness and alterations in cardiac perfusion [3]. This way, AGEs may accelerate arteriosclerosis and impair myocardial contraction. The reduced flexibility of matrix proteins can also result in myocardial rigidity and prolonged repolarisation of the cardiac contraction, which may also induce diastolic dysfunction [3]. However, limited human studies, mainly in selected individuals with chronic heart failure, have investigated the association of serum or plasma AGEs with systolic and diastolic function [3, 10–12]. The studies showed links between AGEs and prevalent heart failure and correlations with diastolic but not systolic ventricular function [10–12]. The few studies on the association of AGEs with left ventricular (LV) systolic and diastolic function in small populations have shown inconsistent findings. One study found correlations between serum AGEs and unfavourable echocardiographic diastolic but not systolic function in individuals with type 1 diabetes free of heart failure [13], while a more recent study showed worse systolic and diastolic function with higher serum AGEs only in participants with normal glucose metabolism [14]. Therefore, further investigation at the population level is essential to further clarify the role of AGEs in heart failure pathophysiology and treatment. This can also make way for further research into the value of AGEs for heart failure risk prediction and primary prevention.

Assessment of the skin autofluorescence (SAF) property of AGEs provides a non-invasive, rapid and valid marker of AGE accumulation in the body [8, 15] and has shown associations with cardiac tissue glycation [16]. Using this method, we examined the association of AGEs measured by SAF with prevalent heart failure in the population-based Rotterdam Study. Next, we investigated the association of AGEs with echocardiographic parameters of LV systolic and diastolic function among individuals free of heart failure. We further examined the impact of type 2 diabetes and possible sex differences in these associations.

## Methods

### Study population

**Fig. 1:**
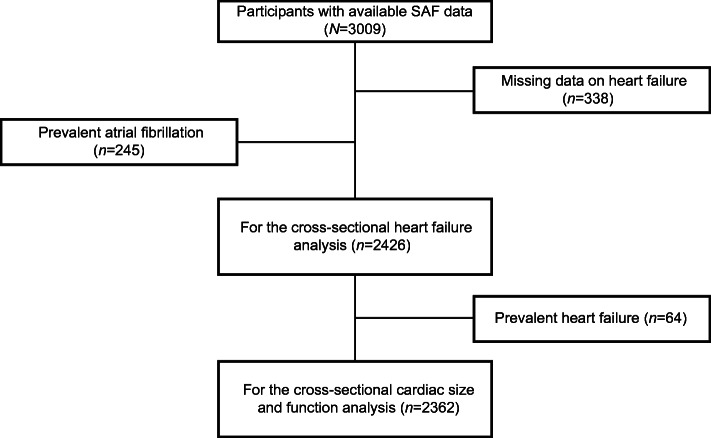
Flowchart of the included study participants

This investigation was conducted within the Rotterdam Study, a prospective cohort initiated in 1989 among 7983 individuals 55 years of age or older (RS-I) who lived in the Ommoord district of Rotterdam, the Netherlands [17]. In 2000, 3011 participants who had reached 55 years or moved to the district since the beginning of the study were included (RS-II). In 2006, a further extension of the cohort was initiated, recruiting 3932 individuals 45–54 years of age (RS-III). Participants with SAF measurements at the sixth examination of the first cohort (RS-I-6), the fourth examination of the second cohort (RS-II-4) and the second examination of the third cohort (RS-III-2) were considered for this study (*N*=3009). After excluding participants without information on heart failure status (*n*=338), we excluded those with atrial fibrillation (*n*=245) to make sure of the quality of the echocardiographs. The remaining 2426 participants underwent analysis of the association between SAF and heart failure. Afterwards, 64 participants with prior diagnosis of heart failure were further excluded for the analysis of the association between SAF and LV function (Fig. 1).

The Rotterdam Study has been approved by the Medical Ethics Committee of Erasmus MC (registration no. MEC02.1015) and the Dutch Ministry of Health, Welfare and Sport (population Screening Act WBO, license no. 1071272-159521-PG). The Rotterdam Study is registered at the Netherlands National Trial Register (NTR; www.trialregister.nl) and the WHO International Clinical Trials Registry Platform (ICTRP; www.who.int/ictrp/network/primary/en/) under catalogue no. NTR6831. All individuals provided written informed consent to participate and have their information obtained from physicians.

### Echocardiography

Rotterdam Study participants underwent transthoracic two-dimensional, M-mode and Doppler echocardiography according to a standard protocol [18]. Echocardiographic examinations were performed using a commercially available device (Vivid I; GE Healthcare, Little Chalfont, UK) with a 3S-RS sector array probe (1.5–3.6 MHz) by the same trained personnel. LV systolic function was assessed using LV ejection fraction (LVEF, %) calculated based on the Teichholz formula [19]. For diastolic function, early transmitral diastolic filling velocity (E wave) and late diastolic filling velocity (A wave) during three cardiac cycles were used to calculate E/A ratio. Left atrial (LA) anteroposterior diameter (mm) and E/e′ ratio, calculated using early diastolic longitudinal filling velocity of the septal mitral annulus (septal e′), were also included. LV mass (LVM) indexed by body surface area (g/m^2^) was calculated using the cube formula.

### Measurement of SAF

SAF was measured at the same examinations rounds as the echocardiographic measurements. The AGE Reader (DiagnOptics, Groningen, the Netherlands) was used to measure SAF, a marker for the amount of AGEs accumulated in the skin, non-invasively and quickly. This method evaluates the fluorescent property of AGEs [15] by quantifying the emission of a specific wavelength of skin autofluorescence after being excited by a specific wavelength of light and has been validated for determining the accumulation of AGEs (pentosidine, carboxymethyl lysine and carboxyethyl lysine) in skin biopsies from the same site as SAF measurement [20]. In practice, around 4 cm^2^ of the skin is illuminated with an excitation light with a peak around 370 nm (300–420 nm) and guarded against external light [15, 20]. The emitted spectrum of AGEs (420–600 nm) is then measured by the AGE Reader. Afterwards, SAF is calculated in arbitrary units (AU) based on light excitation/reflection ratio by AGE Reader software (version 2.3.0) using validated algorithms that account for skin colour with a UV reflectance of 6–10%. In our study, the dominant forearm of participants was placed on the device for three consecutive measurements. Individuals were asked not to use skin creams before measurement. Participants with darker skins were automatically excluded by the AGE Reader if the mean skin reflectance was ≤6%. Then, the mean of three measurements was calculated for a precise SAF value. Extreme values of the three measurements were identified and excluded through a combination of Grubbs’ test and value outside of the mean±4SD scope. In these cases, SAF was calculated as the mean of the two remaining values. Repeated autofluorescence measurements have shown an intra-observer variation of 5–6% within 1 day [20]. In our study, there was an excellent absolute agreement between the SAF measurements in each person within a day (intraclass correlation 0.93 [95% CI 0.92, 0.94] in a random sample in 500 individuals).

### Heart failure assessment

Ascertainment of prevalent and incident heart failure has been explained in detail elsewhere [21]. Prevalent heart failure at entry into the original Rotterdam Study cohort was based on clinical information from medical records for all participants using a validated score, similar to the definition of heart failure by the European Society of Cardiology [22]. Heart failure was defined as a combination of the presence of symptoms or signs of heart failure, confirmed by objective evidence of cardiac dysfunction. Diagnosis was confirmed by a medical specialist. For the subsequent cohorts, at baseline, medical records of participants were screened for evidence of prevalent heart failure. After entry, information on incident heart failure was systematically collected from physician records and verified hospital diagnoses gathered from all hospitals in Rotterdam [21]. Incident date for heart failure was set as the date of the first occurrence of symptoms suggestive of heart failure or the day of receipt of a first prescription for a loop diuretic or an ACE inhibitor, whichever came first. Individuals with prevalent heart failure at study entry and those with incident heart failure events prior to entry to this study were defined as prevalent heart failure.

### Cardiovascular risk factors

Systolic and diastolic BP were measured using a random-zero sphygmomanometer on the right arm of participants when in a resting sitting position. Two measurements were performed and the average of the two was used. Hypertension was defined as a systolic BP ≥140 mmHg, diastolic BP ≥90 mmHg or use of antihypertensive medication. The indication for use of antihypertensive medication was determined by a physician. BMI was calculated based on weight (kg) divided by height squared (m^2^). Waist circumference was measured at the level midway between the lower rib and the iliac crest with participants in a standing position. Data on monthly income, smoking status (never, current and past) were extracted from questionnaires. Physical activity was measured as metabolic equivalent of task in hours per week (METh/week) based on questionnaires. Type 2 diabetes was defined as fasting blood glucose >7.0 mmol/l, or use of glucose-lowering medication. Prevalent CHD was defined as a history of clinically manifest myocardial infarction verified from the medical records [21].

### Statistical analysis

Characteristics of the study population were presented as mean (SD) or median (IQR) for continuous variables and as *n* (%) for dichotomous variables across tertiles of SAF. Missing data on covariates were imputed using multiple imputations (*n*=5).

The cross-sectional association of SAF, on a continuous scale, with prevalent heart failure was assessed using logistic regressions. Next, after excluding individuals with prevalent heart failure, we examined the association of SAF with parameters of cardiac function using linear regressions. Analyses were first adjusted for age and sex (Model 1). We then added waist circumference, hypertension, type 2 diabetes, CHD, physical activity, monthly income and smoking status to the adjustments (Model 2). We checked for interactions between SAF and sex, type 2 diabetes, hypertension and CHD separately, and investigated the possible non-linearity of the association of SAF with outcomes in the multivariate analyses (*p* value of 0.2 was considered significant). Next, all analyses were stratified by sex.

Sensitivity analyses were carried out once after excluding participants with CHD and once replacing waist circumference with BMI in the adjustments. We also assessed the association between skin reflectance and our outcomes. Next, we performed the main analyses once with further adjustment with coffee intake, as a proxy for caffeine intake. In ancillary analyses, we further adjusted for eGFR in a subgroup with available kidney function measurements to assess the role of kidney function. Finally, we repeated the analyses among participants with complete information on covariates.

Analyses and production of figures were performed using statistical software R, version 3.6.1 (accessed 7 May 2019) (https://www.r-project.org/) (Packages: mice, rms, ggplot2).

## Results

Characteristics of participants, per SAF tertile, are shown in Table 1. Participants in higher SAF tertiles were older (mean [SD] 68.7 [9.5], 71.5 [9.6] and 74.9 [9.0] years for the first, second and third tertile, respectively). The mean (SD) SAF in the total population was 2.37 (0.5) AU and SAF had a normal distribution (ESM Fig. 1). Diabetes was more frequent among individuals in higher tertiles of SAF (*n* [%] 76 [9.4], 113 [14.0] and 168 [20.8] for the first, second and third tertile, respectively), as were CHD and hypertension. LVEF was lower in participants in the higher SAF tertile (mean [SD] 68.4% [4.91%] for the first tertile and 66.2% [7.14%] for the third tertile). Participants in higher SAF tertiles also had higher E/e′ and LA diameter but lower E/As; differences were not apparent for LVM index. Finally, prevalent heart failure was more frequent with increasing SAF tertile (*n* [%] 9 [1.1], 14 [1.7] and 41 [5.1] for the first, second and third tertile, respectively).

**Table 1:** Characteristics of participants per tertile of SAF

Variable	Tertile 1 (*n*=809)	Tertile 2 (*n*=809)	Tertile 3 (*n*=808)
Age, years	68.7 (9.5)	71.5 (9.6)	74.9 (9.0)
Women, *n* (%)	561 (69)	457 (57)	390 (48)
Waist circumference, cm	92.5 (12)	93.8 (12.60)	95.8 (13.7)
Hypertension, *n* (%)	521 (64.4)	571 (70.6)	602 (74.5)
Type 2 diabetes, *n* (%)	76 (9.4)	113 (14.0)	168 (20.8)
CHD, *n* (%)	22 (2.7)	41 (5.1)	73 (9.0)
Smoking, *n* (%)			
Never	325 (40.2)	258 (31.9)	210 (26.0)
Current	67 (8.3)	91 (11.2)	135 (16.7)
Former	414 (51.2)	460 (56.9)	462 (57.2)
Income per month, *n* (%)			
<1200 €	18 (2.2)	18 (2.2)	29 (3.6)
1200–2100€	90 (11.1)	134 (16.6)	152 (18.8)
>2100 €	701 (86.7)	657 (81.2)	627 (77.6)
Coffee intake, g/day	320 (204)	315 (196)	338 (214)
Physical activity, MET h/week	61.1 (56.9)	56.2 (48.9)	52.0 (53.1)
eGFR, ml/min per 1.73 m^2^	66.6 (13.6)	65.0 (14.4)	60.5 (14.9)
LVEF, %	68.4 (4.91)	67.5 (5.89)	66.2 (7.14)
E/e′	9.52 (3.64)	9.64 (3.27)	10.08 (3.88)
E/A	0.92 (0.25)	0.91 (0.27)	0.87 (0.30)
LA diameter, mm	40.9 (4.96)	41.5 (5.72)	42.5 (5.88)
LVM index, g/m^2^	71 (17.10)	72 (17.86)	72 (19.51)
SAF, AU	1.88 (0.18)	2.31 (0.11)	2.94 (0.39)
Skin reflectance, %	16 (5.00)	16 (5.15)	16 (5.55)
Prevalent heart failure, *n* (%)	9 (1.1)	14 (1.7)	41 (5.1)

Data are presented as mean (SD) or median (IQR) for continuous variables or as *n* (%) for dichotomous variables

The number of individuals with available data on outcomes was 2328 for LVEF, 2295 for E/e′, 2307 for E/A, 2343 for LA diameter and 1181 for LVM index. Data are based on the original dataset. Missing data on covariates (%): hypertension 0.04; waist circumference 0.04; BMI 0.08; smoking 0.17; CHD 0.34; and physical activity 4.1%

The OR for one unit increase in SAF in association with prevalent heart failure was 2.90 (95% CI 1.80, 4.62) after adjustments. We did not find interactions between SAF, age, type 2 diabetes, hypertension or CHD (*p*_interaction_>0.2) in this analysis. After stratifying by sex (OR_interaction_ 1.91 [95% CI 0.91, 5.55], *p*=0.08), the OR of heart failure with one unit increase in SAF was 1.81 (95% CI 0.89, 3.36) among men and 4.16 (95% CI 2.13, 8.16) among women.

Among individuals free of prevalent heart failure (*n*=2362), higher SAF levels showed an inverse association with LVEF (β −0.98% [95% CI −1.45%, −0.50%] for each unit increase in SAF) in Model 2 (Table 2). SAF was also accompanied by higher E/e′ and E/A and by larger LA diameter but the associations were not statistically significant. In multivariate sex-stratified analyses, one unit increase in SAF value was associated with 1.62% lower LVEF (95% CI −2.41%, −0.83%) and 0.44% (95% CI 0.03%, 0.85%) higher E/e′ in men (ESM Table 1).

**Table 2:** Association of SAF with echocardiographic parameters and LV systolic and diastolic function

Outcome	β (95%CI)
LVEF	
Model 1^a^	−1.14 (−1.61, –0.67)
Model 2^b^	−0.98 (−1.45, −0.50)
E/e′	
Model 1^a^	0.24 (−0.05, 0.54)
Model 2^b^	0.13 (−0.17, 0.43)
E/A	
Model 1^a^	0.01 (−0.01, 0.03)
Model 2^b^	0.01 (−0.02, 0.03)
LA diameter	
Model 1^a^	0.26 (−0.19, 0.70)
Model 2^b^	0.07 (−0.36, 0.50)
LVM index	
Model 1^a^	−1.26 (−3.25, 0.72)
Model 2^b^	−1.24 (−3.24, 0.77)

Mean difference (β) (95% CI) for one unit increase in SAF was calculated using linear regression

^a^Model 1 adjusted for age and sex

^b^Model 2 adjusted for age, sex, CHD, type 2 diabetes, hypertension, smoking status, waist circumference, monthly income and physical activity

The number of individuals with available data on outcomes was 2328 for LVEF, 2295 for E/e′, 2307 for E/A, 2343 for LA diameter and 1181 for LVM index

**Fig. 2:**
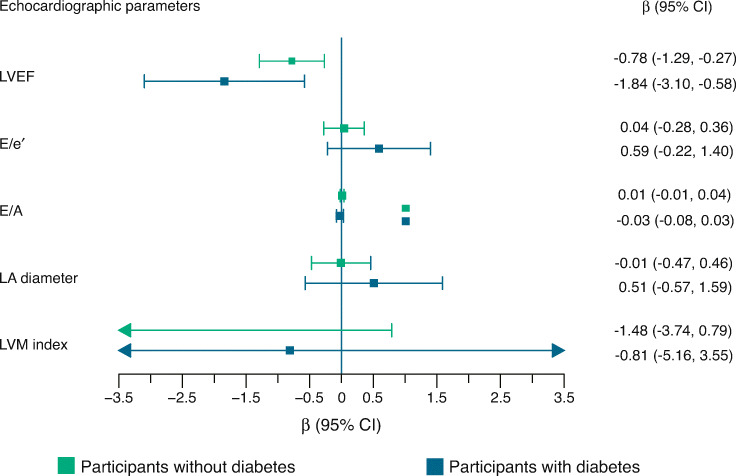
Association of SAF with parameters of LV systolic and diastolic function by type 2 diabetes status. Mean difference (β) (95% CI) for one unit increase in SAF was calculated using linear regressions adjusted for age, sex, CHD, hypertension, smoking status, waist circumference, monthly income and physical activity. The number of participants without diabetes was 2069 and with diabetes was 357 in the original data. Arrows in the figure indicate that CI interval exceeds the width of the *x*-axis

We found significant interactions with type 2 diabetes for the association of SAF with LVEF and E/e′ (*p*_interaction_=0.02 and 0.05, respectively). In multivariate analysis, one unit increase in SAF was associated with 0.78% lower LVEF among participants without diabetes (β −0.78% [95% CI −1.29%, −0.27%]) and with 1.84% lower LVEF among participants with diabetes (β −1.84% [95% CI −3.10%, −0.58%]) (Fig. 2). In the analyses stratified by sex, corresponding results were −2.40% (95% CI −4.57%, −0.24%) in men without diabetes and −1.49% (95% CI −2.35%, −0.64%) in men with diabetes (ESM Fig. 2). Among women, increasing SAF was linked to worse mean LVEF in women with diabetes than in those without diabetes, although the association was not significant. The association between SAF and E/e′ was larger in magnitude among participants with diabetes (Fig. 2), specifically in men with diabetes (β 1.85 [95% CI 0.55, 3.15]) (ESM Fig. 2). We did not find significant associations between SAF and LVM index.

**Fig. 3:**
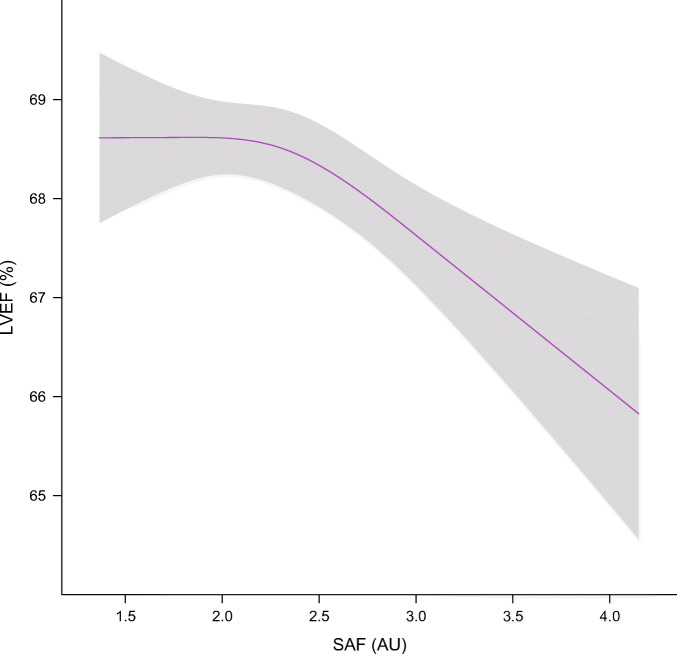
Levels of LVEF in association with SAF levels. The number of individuals with available data on LVEF and SAF was 2328 in the original data

When investigating possible non-linearity in the association of SAF with heart failure and cardiac function parameters in the multivariate adjusted models, we observed a non-linear association with LVEF, resulting in extended worsening of LVEF at higher SAF levels (Fig. 3). We did not observe non-linear associations of SAF with prevalent heart failure or other cardiac function parameters.

In sensitivity analyses, the exclusion of 123 participants with prevalent CHD from our analyses did not substantially change our results (ESM Tables 2, 3). Moreover, adjustment for BMI instead of waist circumference and also performing analyses among participants with complete information on all covariates without imputation yielded similar results (data not shown).

Additionally, we did not find associations between skin reflectance and either of the outcomes adjusted for age and sex; further adjustment of analyses for coffee intake did not change our results drastically (data not shown). We also repeated analyses once with additional adjustment for eGFR in a subgroup with available eGFR measurement (*n*=1137 with heart failure and 1085 excluding heart failure). Here, the association between SAF and heart failure in the total population was attenuated (OR 0.28 [95% CI 0.04, 1.57]). Higher SAF levels maintained its significant association only with lower LVEF (β −0.70% [95% CI −1.36%, −0.05%]) (ESM Table 4). After stratifying these analyses by eGFR, associations between SAF and worsening of LVEF, higher E/e′ and larger LA diameter were stronger in the overall population (and among men) than those with eGFR below 60 ml/min per 1.73 m^2^, despite statistical insignificance (ESM Table 5).

## Discussion

In this population-based study, higher SAF levels were associated with prevalent heart failure. Among participants free of clinical heart failure and, regardless of diabetes status, higher levels of SAF were accompanied by worse LV systolic function while the association with diastolic function was less apparent. The sex-stratified analyses suggested an association between SAF and worse diastolic function that was more prominent in men with diabetes.

AGEs are proposed targets in the treatment of heart failure [3]. However, their role in the pathophysiology of cardiac function and heart failure has been mostly studied in individuals with chronic heart failure [3, 10–12], diabetes or CHD [13, 23]. In our study, higher SAF levels were associated with heart failure regardless of diabetes or CHD status. In addition, among individuals without heart failure, higher levels of SAF were associated with worse LV systolic function. AGEs may adversely affect LV systolic function by accelerating arteriosclerosis. They induce thrombosis and increase vasoconstriction [3]. Further, by cross-linking with LDL, AGEs reduce their clearance and make these particles more atherogenic [3]. They may also impair myocardial contraction by reduction of intracellular calcium leading to decreased myocardial contractility [24]. On the other hand, excessive cross-linking as a result of AGE accumulation can reduce matrix flexibility and increase cellular rigidity in the heart [3]. Impaired relaxation due to the same reduction of intracellular calcium and prolonged repolarisation may also add to this. This way, AGE accumulation is also suspected to result in diastolic dysfunction [3, 25].

In our study, SAF was linked to worse LV function, especially systolic function, irrespective of diabetes status. Until now, one study in 52 individuals with type 1 diabetes has shown correlations between serum AGEs and echocardiographic relaxation time and LV diameter during diastole but not fractional shortening or LVEF [13]. However, a more recent study found associations between serum AGEs and worse systolic and diastolic function among 280 participants with normal glucose levels but not among 242 participants with type 2 diabetes [14], questioning whether serum AGEs could be representative of myocardial AGE accumulation. In contrast, we showed that the higher levels of SAF was accompanied by worse LV function regardless of diabetes status, although this association was more prominent in participants with diabetes which could indeed be due to enhanced myocardial accumulation of AGEs and more evident diastolic dysfunction in diabetes [3, 26]. The more prominent associations with poorer indexes of diastolic dysfunction in those with diabetes emphasise this idea. Our observations among participants without diabetes, in line with previous reports [14], highlight the extensive involvement of AGEs in heart failure pathophysiology. AGE accumulation is not limited to type 2 diabetes and is also enhanced in other states of oxidative stress, increased intake or intestinal formation in response to sweetened foods [27]. However, the stronger tendency to have poor systolic function with higher SAF values within this group who have lower AGE accumulation may be due to a higher susceptibility of pathways related to arteriosclerosis to small increases in AGEs.

We also report possible sex differences in the association of AGEs with heart failure and LV function. Higher levels of SAF showed a stronger association with prevalent heart failure in women. This is while mean SAF levels were lower among women than men, in line with other studies conducted in a Dutch population [28]. Heart failure occurs more frequently and earlier among men [29]. Men also have a higher heart failure mortality risk [29]. Thus, it is possible that men with severe heart failure and higher levels of AGEs might have not been captured in the current sample due to earlier mortality (survival bias). However, the association between higher SAF levels and worse systolic function was more pronounced in men and, despite insignificance, the associations between SAF and poor LV diastolic function were stronger in men. Notably, SAF was accompanied by higher E/e′, a direct measure of diastolic function, in men. The correlation between myocardial fibrosis and the severity of diastolic dysfunction could have resulted in this observation [30]. However, excluding participants with prevalent CHD did not change our results. While these results warrant further replication, the smaller associations found in women may simply be due to lower SAF levels measured in women. However, the significant association with heart failure in women, even with lower SAF values, may point towards potential sex-based differences in heart failure pathophysiology and the contribution of AGEs to those mechanisms. In the context of heart failure, men are more prone to macrovascular coronary artery disease and myocardial infarction, while microvascular dysfunction/endothelial inflammation are key players in heart failure with preserved LVEF, which is more prominent in women. Thus, AGEs may have a stronger bond with endothelial inflammation–coronary microvascular dysfunction in women and with cardiac and macrovascular function in men; mechanisms which lead to heart failure. Previous studies suggest that cardiometabolic susceptibility is increased by androgens (which drive microvascular dysfunction) through AGE interference in women and that reduction in cell viability by AGE-induced damage by physiological increase in testosterone occurs [31, 32]. The cross-sectional nature of our study and the overlap in the effect estimates of the associations for men and women warrants replication of our results in prospective settings before further inferring sex differences regarding the implications of AGEs in the pathophysiology of heart failure.

Higher caffeine intake has previously shown associations with increased SAF and higher risk of heart failure [33–35]. However, further adjustment of analyses for coffee intake, as a major source of caffeine, did not affect our results. In a smaller subgroup, adjustment for kidney function attenuated the associations. AGE accumulation is expected to increase in individuals with renal dysfunction due to decreased clearance and enhanced oxidative stress [25]. This is while some AGEs, like MG-H1 and Glo1 might also have a role in the development of kidney disease [36, 37]. The bidirectional association of renal and cardiac function in the context of cardiorenal syndrome makes the situation more complex [38]. This way, further adjustment by eGFR status in our study could have resulted in collider bias [39]. Regardless, the substantial attenuation of our results suggests a mediating role for the kidney in the association between AGEs and heart failure [25]. However, we did not find distinct differences in the associations between SAF and LV function among those with and without reduced kidney function, possibly due to insufficient power.

Use of AGE-crosslink breakers in heart failure has produced inconclusive findings. While they improved cardiac function among 23 individuals with heart failure and LVEF>50% [3], the BENEFICIAL trial did not report improvement in systolic or diastolic function among 102 individuals with heart failure and LVEF<40% [40]. Based on our findings, population selection based on LVEF might have resulted in the contrasts between studies. The weaker association with diastolic function in our study also highlights pathways other than cross-linkage connecting AGEs to cardiac function that should be taken into account. Therefore, further research on the role of AGEs in heart failure may assist in development of effective therapies. Our results also merit considering sex differences in this context.

Strengths of our study include access to a detailed and well-defined cohort from the general population, meticulous adjudication of heart failure events and use of a non-invasive method to measure AGEs in skin biopsies [41]. The echocardiography data was collected using a standardised protocol by trained echocardiographers with good inter-reader and intra-reader agreement. Tissue AGE accumulation may be more relevant to tissue damage than plasma levels [20]. SAF measurement in the skin is a convenient method that provides a non-invasive, quick and automated alternative to invasive methods for assessment of conditions related to AGE accumulation. Measured AGE-related SAF levels have shown an association with cardiac tissue glycation [16, 20]. There were also limitations. First, we could not study the link between SAF and incident heart failure due to insufficient follow-up data, since SAF was measured in the latter examinations of the Rotterdam Study and the cross-sectional nature of our study could limit the inferences. Second, we could not exclude bias due to unmeasured confounding and survival bias. Comparing the included sample with the excluded (*n*=538) individuals, to address possible selection bias, the included sample was slightly younger and appeared healthier (a lower proportion of included individuals had diabetes, CHD and heart failure and included individuals had higher mean LVEF and better diastolic function parameters) (ESM Table 6). Moreover, mean SAF levels were lower in the included sample. Third, a weakness of SAF is that MG-H1 and carboxymethyl lysine are not fluorescent. MG-H1 is a major contributor to LV remodelling after myocardial infarction [23]. Therefore, our findings may underestimate the link between AGEs and cardiac function. Fourth, LVEF was calculated using M-mode measurements using the Teichholz formula, which can overestimate LVEF in the case of wall motion abnormalities [19]. There was a moderate degree of agreement between the calculated and biplane method in a sample of participants (intraclass correlation 0.69 [95% CI 0.59, 0.77]). Moreover, a subgroup of individuals had available eGFR measurement since serum creatinine was not measured in the Rotterdam Study visits included in the current study. Fifth, the AGE Reader may not make measurements in very dark skin. Most of the participants in our study were of Dutch descent and had lighter skin colour, as previously reported (very white [3.4%], white [79.1%], white to olive [14.5%], light brown [1.7%], brown [1.1%], dark brown/black [0.2%]) [6]. This suggests that only a minimal proportion of participants might have been excluded because of very dark skin colour. Regardless, this could limit the generalisability of our study. Thus our observations should be generalised with caution. Finally, the *NAT2* gene has been associated with SAF levels [42]. The SNP tagging the faster metabolising *N*-acetyltransferase enzyme has been associated with a lower SAF value. Future studies are warranted to study a possible association of *NAT2* with heart failure and cardiac dysfunction.

### Conclusions

Higher levels of SAF were associated with prevalent heart failure irrespective of diabetes status. Among individuals free of heart failure, higher SAF levels were associated with poorer LV systolic and diastolic function, although the associations were more prominent for systolic function. Sex-stratified analyses suggested an inverse association between AGEs and parameters of diastolic function that was more prominent in men with diabetes. Further prospective research on the mechanisms linking AGEs to heart failure is warranted.

## Supplementary information

ESM (PDF 713 kb)

## Data Availability

The data underlying this article are not publicly available due to legal and ethical restraints. Sharing of individual participant data was not included in the informed consent of the study and there is potential risk of revealing participant identities as it is not possible to completely anonymise the data. However, data are available from the corresponding author on reasonable request.

## Abbreviations

AGE: Advanced glycation end-product
AU: Arbitrary units
LA: Left atrial
LV: Left ventricular
LVEF: LV ejection fraction
LVM: LV mass
METh: Metabolic equivalent of task in hours
RS: Rotterdam Study
SAF: Skin autofluorescence
