# Supplementary material for: Advanced glycation end-products, cardiac function and heart failure in the general population: The Rotterdam Study
Source: Diabetologia. 2022 Nov 8;66(3):472–81. doi: 10.1007/s00125-022-05821-3 (PMC9892093; doi:10.1007/s00125-022-05821-3)
Supplement: Supplementary file 1 — (PDF 713 kb) [file 125_2022_5821_MOESM1_ESM.pdf]

Electronic Supplementary Material (ESM)

	Page
**ESM Table 1** Association of skin autofluorescence (SAF) with echocardiographic parameters of left ventricular systolic and diastolic function in men and women	2
**ESM Table 2** Association of skin autofluorescence (SAF) with prevalent heart failure, excluding participants with coronary heart disease	4
**ESM Table 3** Association of skin autofluorescence (SAF) with echocardiographic parameters of left ventricular systolic and diastolic function, excluding participants with coronary heart disease	5
**ESM Table 4** Association of skin autofluorescence (SAF) with echocardiographic parameters of left ventricular systolic and diastolic function after further adjustment for kidney function	6
**ESM Table 5** Association of skin autofluorescence (SAF) with echocardiographic parameters of left ventricular systolic and diastolic function stratified by kidney function	7
**ESM Table 6** Comparison of the included study population and excluded participants	8
**ESM Fig. 1** Distribution of skin autofluorescence (SAF) values in the study population	10
**ESM Fig. 2** Association of skin autofluorescence (SAF) with parameters of left ventricular systolic and diastolic function in men and women by diabetes status	11

ESM Table 1 Association of skin autofluorescence (SAF) with echocardiographic parameters of left ventricular systolic and diastolic function in men and women

Outcomes		Men β (95%CI)	Women β (95%CI)
LVEF			
	Model 1[a]	−1.75 (−2.54, −0.97)	−0.54(−1.11, 0.03)
	Model 2[b]	−1.62 (−2.41, −0.83)	−0.41 (−0.99, 0.17)
E/e′			
	Model 1[a]	0.52 (0.12, 0.92)	0.06 (−0.36, 0.48)
	Model 2[b]	0.44 (0.03, 0.85)	−0.09 (−0.52, 0.34)
E/A			
	Model 1[a]	0.02 (−0.02, 0.05)	0.001 (−0.03, 0.03)
	Model 2[b]	0.02 (−0.02, 0.05)	−0.003 (−0.03, 0.03)
LA diameter			
	Model 1[a]	0.67 (−0.01, 1.35)	−0.04 (−0.63, 0.56)
	Model 2[b]	0.39 (−0.27, 1.05)	−0.16 (−0.72, 0.40)
LVM index			
	Model 1[a]	0.11 (−2.95, 3.16)	−2.16 (−4.77, 0.45)
	Model 2[b]	−0.29 (−3.44, 2.85)	−1.91 (−4.50, 0.69)

Mean differences (β) (95% CI) for one unit increase in SAF were calculated using linear regressions

Model 1 adjusted for age and sex (in the overall population)

Model 2 adjusted for age, sex (in the overall population), CHD, type 2 diabetes, hypertension, smoking status, waist circumference, monthly income and physical activity

The number of individuals with available data on outcomes in the overall population was 2328 for LVEF, 2295 for E/e′, 2295 for E/e′, 2307 for E/A, 2343 for LA diameter and 1181 for LVM index

LVEF: Left ventricular ejection fraction, E/e′: E wave/ septal e′ ratio, E/A; mitral E wave/ A wave ratio, LA diameter: left atrial diameter, LVM index; left ventricular mass index

ESM Table 2 Association of skin autofluorescence (SAF) with prevalent heart failure, excluding participants with coronary heart disease

	Overall population OR (95% CI)	Men OR (95% CI)	Women OR (95% CI)
Model 1[a]	2.76 (1.69, 4.35)	1.81 (1.68, 8.37)	4.02 (2.02, 7.71)
Model 2[b]	2.81 (1.65, 4.73)	1.60 (0.69, 3.56)	4.39 (2.15, 8.97)

Odds ratios (OR) (95% CI) of prevalent heart failure for one unit increase in SAF were calculated using logistic regressions

[a]Model 1: adjusted for age, sex (in the overall population)

[b]Model 2 adjusted for age, sex (in the overall population), CHD, type 2 diabetes, hypertension, smoking status, waist circumference, monthly income and physical activity

The number of individuals in the analyses were 2426 in the overall population, 1018 men and 1408 women

ESM Table 3 Association of skin autofluorescence (SAF) with echocardiographic parameters of left ventricular systolic and diastolic function, excluding participants with coronary heart disease

outcomes	Overall population β (95%CI)	Men β (95%CI)	Women β (95%CI)
LVEF	-1.03 (-1.49, -0.56)	-1.58 (-2.36, -0.79)	-0.57 (-1.15, 0.01)
E/e′	0.03 (-0.28, 0.34)	0.30 (-0.12, 0.73)	-0.14 (-0.57, 0.30)
E/A	0.004 (-0.02, 0.03)	0.02 (-0.01, 0.06)	-0.01 (-0.04, 0.02)
LA diameter	0.07 (-0.37, 0.51)	0.55 (-0.17, 1.26)	-0.22 (-0.79, 0.35)
LVM index	-1.54 (-3.62, 0.54)	-0.81 (-4.20, 2.58)	-2.01 (-4.65, 0.63)

Mean difference (β) (95% CI) for one unit increase in SAF was calculated using linear regressions adjusted for age, sex (in the overall population), hypertension, smoking status, waist circumference, monthly income and physical activity

LVEF: Left ventricular ejection fraction, E/e′: E wave/ septal e′ ratio, E/A; mitral E wave/ A wave ratio, LA diameter: left atrial diameter, LVM index; left ventricular mass index

ESM Table 4 Association of skin autofluorescence (SAF) with echocardiographic parameters of left ventricular systolic and diastolic function after further adjustment for kidney function*

	Overall population	Men	Women
outcomes	β (95%CI)	β (95%CI)	β (95%CI)
LVEF	-0.70 (-1.36, -0.05)	-0.79 (-1.88, 0.30)	-0.60 (-1.41, 0.21)
E/e'	0.14 (-0.20, 0.49)	0.30 (-0.21, 0.80)	0.06 (-0.41, 0.53)
E/A	-0.01 (-0.04, 0.03)	-0.01 (-0.06, 0.05)	-0.01 (-0.06, 0.04)
LA diameter	-0.10 (-0.75, 0.54)	0.39 (-0.63, 1.41)	-0.53 (-1.37, 0.31)

Mean differences (β) (95% CI) for one unit increase in SAF were calculated using linear regressions adjusted for age, sex (in the overall population), CHD, hypertension, smoking status, waist circumference, monthly income and physical activity and eGFR

*The number of participants with available eGFR values for these analyses was 1085 (466 men and 619 women)

LVEF: Left ventricular ejection fraction, E/e': E wave/ septal e' ratio, E/A; mitral E wave/ A wave ratio, LA diameter: left atrial diameter, eGFR estimated glomerular filtration rate

ESM Table 5 Association of skin autofluorescence (SAF) with echocardiographic parameters of left ventricular systolic and diastolic function stratified by kidney function

		eGFR<60	
	Overall population	Men	Women
outcomes	β (95%CI)	β (95%CI)	β (95%CI)
LVEF	-0.89 (-2.04, 0.26)	-0.49 (-1.91, 0.92)	-1.44 (-3.63, 0.75)
E/e′	0.43 (-0.12, 0.99)	0.52 (-0.15, 1.19)	-0.002 (-0.12, 0.12)
E/A	-0.01 (-0.07, 0.05)	0.003 (-0.07, 0.08)	-0.02 (-0.12, 0.09)
LA diameter	0.04 (-1.04, 1.12)	0.48 (-0.82, 1.79)	-1.22 (-3.31, 0.86)
		eGFR>=60	
LVEF	-0.44 (-1.24, 0.37)	-0.44 (-1.24, 0.37)	-0.18 (-1.07, 0.71)
E/e′	-0.002 (-0.45, 0.44)	-0.002 (-0.45, 0.44)	0.02 (-0.51, 0.55)
E/A	-0.01 (-0.06, 0.04)	-0.01 (-0.06, 0.04)	-0.02 (-0.07, 0.04)
LA diameter	-0.17 (-0.99, 0.65)	-0.17 (-0.99, 0.65)	-0.37 (-1.31, 0.57)

Mean differences (β) (95% CI) for one unit increase in SAF were calculated using linear regressions adjusted for age, sex (in the overall population), CHD, hypertension, smoking status, waist circumference, monthly income and physical activity

*The number of participants with eGFR<60 ml/min per 1.73 m^2 was 428 and with eGFR>=60 ml/min per 1.73 m^2 was 663

LVEF: Left ventricular ejection fraction, E/e′: E wave/ septal e′ ratio, E/A; mitral E wave/ A wave ratio, LA diameter: left atrial diameter, LVM index; left ventricular mass index, eGFR estimated glomerular filtration rate

ESM Table 6 Comparison of the included study population and excluded participants

Variable	Excluded (*n*= 538)	Included (*n*= 2426)
Age, years	77.1 (6.2)	71.9 (9.7)
Women, *n* (%)	290 (50)	1408 (58)
Waist circumference, cm	97 (13)	94 (13)
Hypertension, *n* (%)	478 (82.6)	1694 (69.9)
Type 2 diabetes, *n* (%)	38 (18.4)	357 (14.7)
CHD, *n* (%)	35 (17.0)	136 (5.6)
Smoking, *n* (%)		
Never	199 (34.4)	793 (32.7)
Current	38 (6.6)	293 (12.1)
Former	342 (59.1)	1336 (55.2)
Income, *n* (%)		
<1200 €	13 (2.2)	65 (2.7)
1200–2100 €	81 (13.9)	376 (15.5)
>2100 €	489 (83.9)	1985 (81.8)
Physical activity , METh/week	49.34 (47.2)	56.44 (53.2)
eGFR, ml/min per 1.73 m^2	55.5 (15.6)	64.6 (14.4)
LVEF, %	63.8 (8.93)	67.4 (6.12)
E/e′	11.93 (5.59)	9.75 (3.61)
E/A	1.30 (0.86)	0.90 (0.27)
LA diameter, mm	47.78 (7.77)	41.62 (5.57)
LVM index, g/m^2	76.95 (20.04)	71.73 (18.37)
SAF, AU	2.53 (0.49)	2.38 (0.51)
Prevalent heart failure, *n* (%)	45 (21.7)	64 (2.6)

Data are presented as mean (SD) or median (IQR) for continuous variables or as *n* (%) for dichotomous variables

ESM Fig. 1 Distribution of skin autofluorescence (SAF) values in the overall study population

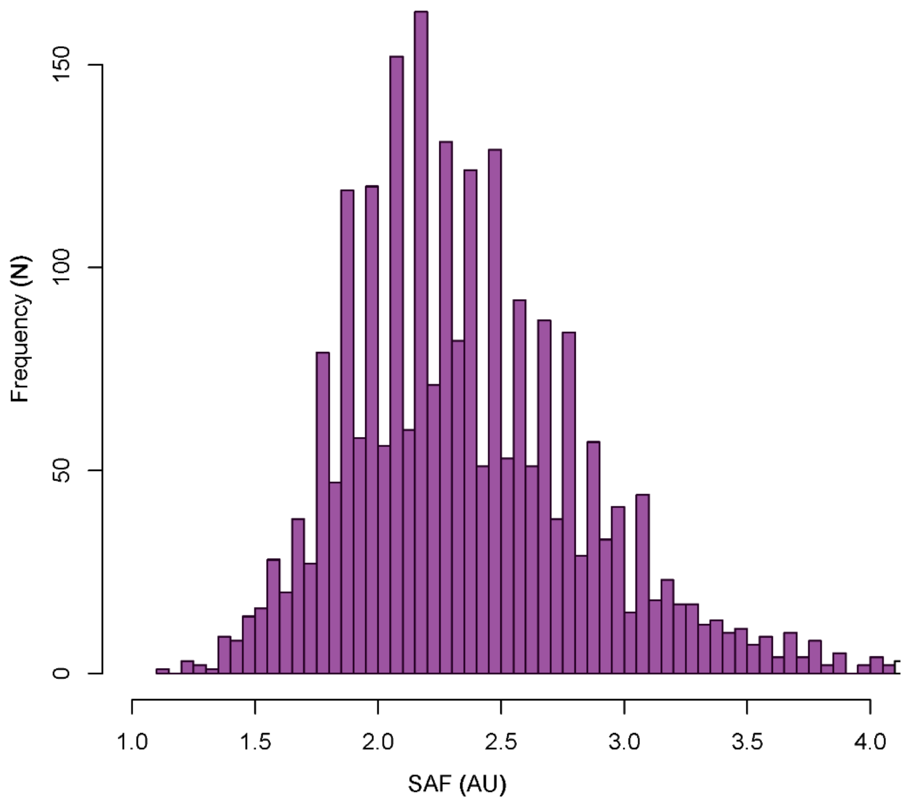

ESM Fig. 2 Association of skin autofluorescence (SAF) with parameters of left ventricular systolic and diastolic function in men and women by diabetes status

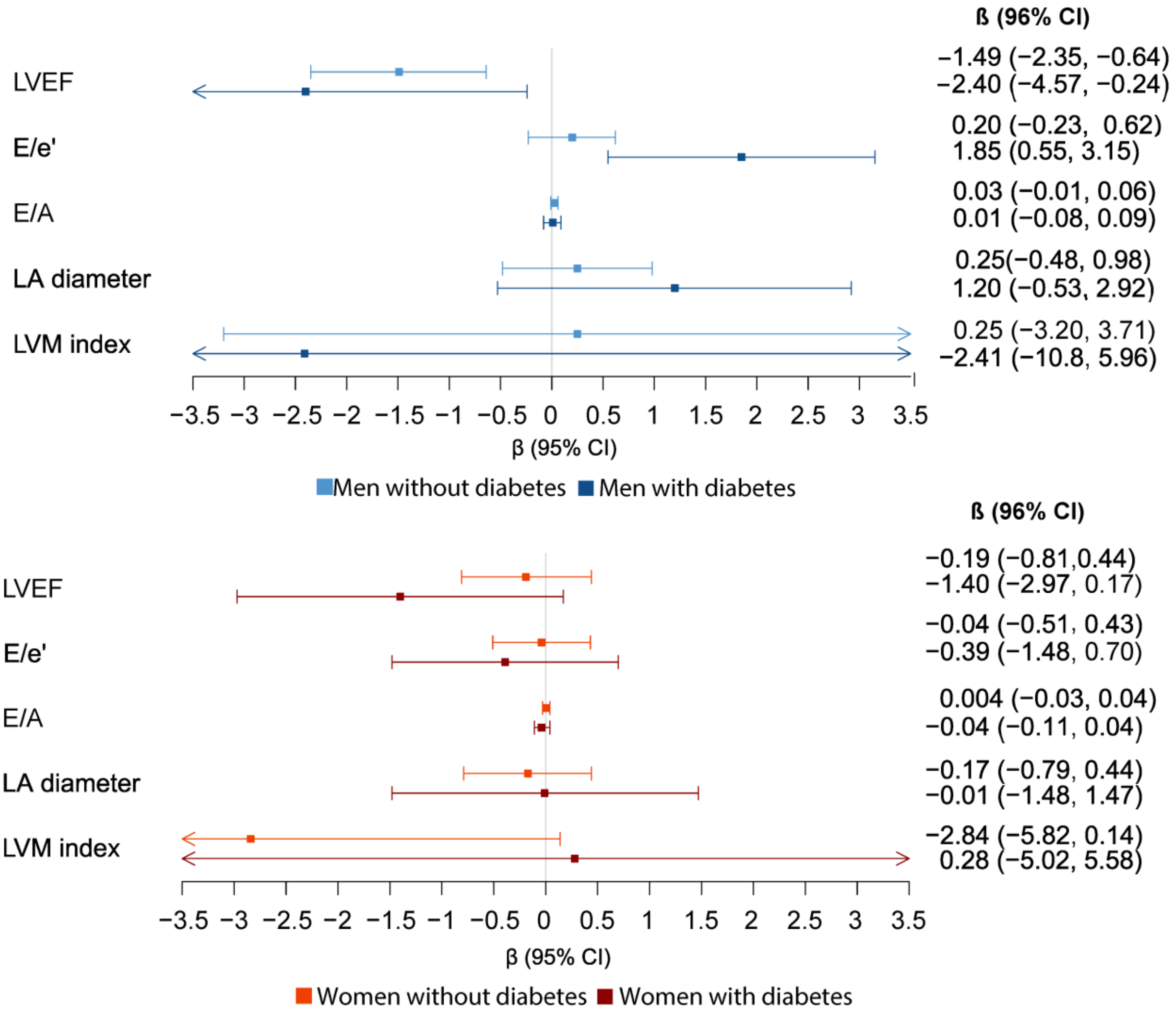

Mean differences (β) (95% CI) for one unit increase in SAF were calculated using linear regressions adjusted for age, CHD, hypertension, smoking status, waist circumference, monthly income and physical activity

The number of men with and without diabetes in the analyses was 171 and 783, respectively

The number of women with and without diabetes in the analyses was 186 and 1222, respectively

LVEF: Left ventricular ejection fraction, E/e’: E wave/ septal e’ ratio, E/A; mitral E wave/A wave ratio, LA diameter: left atrial diameter, LVM index; left ventricular mass index
